# “Because it is a rare disease…it needs to be brought to attention that there are things out of the norm”: a qualitative study of patient and physician experiences of Wilson disease diagnosis and management in the US

**DOI:** 10.1186/s13023-023-02778-3

**Published:** 2023-06-22

**Authors:** Karen M Bailey, Navdeep Sahota, Uyen To, Peter Hedera

**Affiliations:** 1 OPEN Health, London, UK; 2 Yale University, New Haven, Connecticut, United States; 3 University of Louisville, Louisville, KY USA; 4 Department of Neurology Institution, University of Louisville, 220 Abraham Flexner Way, Suite 606, Louisville, KY 40202 USA

**Keywords:** Wilson disease, Qualitative, Interviews, Patient-reported, Treatment experience

## Abstract

**Background:**

Wilson disease (WD) is a genetic disorder of copper metabolism that leads to copper accumulation in various organs, primarily the liver and brain, resulting in heterogenous hepatic, neurologic, and psychiatric symptoms. Diagnosis can occur at any age, requiring lifelong treatment, which can involve liver transplantation. This qualitative study aims to understand the wider patient and physician experience of the diagnosis and management of WD in the US.

**Methods:**

Primary data were collected from 1:1 semi structured interviews with US-based patients and physicians and thematically analyzed with NVivo.

**Results:**

Twelve WD patients and 7 specialist WD physicians (hepatologists and neurologists) were interviewed. Analysis of the interviews revealed 18 themes, which were organized into 5 overarching categories: (1) Diagnosis journey, (2) Multidisciplinary approach, (3) Medication, (4) The role of insurance, and (5) Education, awareness, and support. Patients who presented with psychiatric or neurological symptoms reported longer diagnostic journeys (range 1 to 16 years) than those presenting with hepatic symptoms or through genetic screening (range 2 weeks to 3 years). All were also affected by geographical proximity to WD specialists and access to comprehensive insurance. Exploratory testing was often burdensome for patients, but receipt of a definitive diagnosis led to relief for some. Physicians emphasized the importance of multidisciplinary teams beyond hepatology, neurology, and psychiatry and recommended a combination of chelation, zinc, and a low-copper diet; however, only half the patients in this sample were on a chelator, and some struggled to access prescription zinc due to insurance issues. Caregivers often advocated for and supported adolescents with their medication and dietary regimen. Patients and physicians recommended more education and awareness for the healthcare community.

**Conclusions:**

WD requires the coordination of care and medication among several specialists due to its complex nature, but many patients do not have access to multiple specialties due to geographical or insurance barriers. Because some patients cannot be treated in Centers of Excellence, easy access to reliable and up-to-date information is important to empower physicians, patients, and their caregivers in managing the condition, along with general community outreach programs.

## Background

Wilson disease (WD) is a genetic disorder of copper metabolism that leads to copper accumulation in various organs, primarily the liver and brain. Prevalence ranges from 1 to 30,000 to 1 in 60,000 worldwide, but this figure is currently unknown for the US [1–4]. WD’s complex and variable symptom presentation can result in delayed diagnosis and consequently poorer outcomes [5]. Symptoms include hepatic and neuropsychiatric manifestations [6], with neurological symptoms representing the most frequent clinical WD symptoms [7]. Diagnosis can occur at any age and requires lifelong treatment, which can involve liver transplantation. Genetic treatments are currently being investigated in preclinical studies and clinical trials.

Current guidelines from the American Association for the Study of Liver Diseases (AASLD) [8], the European Association for the Study of the Liver (EASL) [2], and the European Society for Paediatric Gastroenterology Hepatology and Nutrition (ESPGHAN) [1] provide a general consensus on WD treatment. The first line of treatment is a chelating agent to remove copper from the body, usually via administration of oral d-penicillamine or trientine. Following this initial treatment, for some with WD, disease symptoms can be managed with zinc therapy as an alternative to chelating agents, along with a low-copper diet. Up until recently, detailed guidance on multidisciplinary management for WD was not provided. However, the recent AASLD guidance update emphasizes this type of management approach [8]. Patients can also experience fertility issues due to hormonal irregularities occurring with WD and challenges with pregnancy with adverse outcomes, such as miscarriage, more frequently seen in untreated women [9–11]. The literature is conflicting in terms of the continued use of copper chelation medications during pregnancy [9, 11], however current guidelines support their reduction [8].

Previous research reviewing medical records in India and the United Kingdom found up to one-third of patients experienced misdiagnosis, concluding that due to the rarity of the disease and lack of clinician knowledge, patients with WD struggle to get access to medical attention and thus a delay occurs before diagnosis [12, 13]. There is also a deficit of up-to-date qualitative data on the patient-reported experience and outcomes of WD, particularly in the US, where there are 7 Wilson Disease Association–designated Centers of Excellence (COEs), but their limited number means only a small percentage of patients can access them.

To address the data gap, the aim of this qualitative study was to understand the experience of the diagnosis and management of WD in the US, including the role of multidisciplinary care teams, from the perspectives of patients and from physicians in the hepatology, and neurology specialties.

## Results

### Participant characteristics

Table 1 shows patient participant demographics. Out of the 12 patients interviewed, 58% (n = 7) were women. The average age of participants was 40 years, and the average age at diagnosis was 23 years (22.75, range 6–48 years). Most of the sample were White (n = 10), and an estimated 20% of the sample were being treated in a COE. Patient quotes are labeled with ID number, gender, and age.

**Table 1:** Patient participant characteristics (n = 12)

Age, years	
Average (range)	40 (19–72)
**Age at diagnosis (years)**	
Average (range)	22.75 (6–48)
**Gender**, n (%)	
Men	5 (42)
Women	7(58)
**Ethnicity**	
White	10 (83)
Hispanic	1(8)
Native American	1(8)

Seven physicians were interviewed: 4 hepatologists (2 of whom were pediatric hepatologists) and 3 neurologists. Most physicians (5/7) were based in a COE specializing in WD, and the remainder were based at academic medical centers and transplant units. On average, the physicians saw 12 (11.57) patients with WD in the last year (Table 2). Physician quotes are labeled with ID number and discipline.

**Table 2:** Physician participant characteristics

Specialty, n (%)	
Pediatric hepatologist	1 (14)
Hepatologist	2 (29)
Neurologist	3 (43)
Pediatric transplant hepatologist	1 (14)
**Total**	7 (100)
**No. of patients with WD seen in the last year**	
Average (range)	11.57 (3–24)

## Categories and themes

The analysis resulted in 5 overarching categories, each comprising a set of main themes (n = 18) and subthemes (n = 23) (Table 3).

**Table 3:** – Categories, themes, and subthemes

Category 1: Diagnosis journey												
Theme 1: Early presentation			Theme 2: Physician involvement & communication				Theme 3: Extensive testing				Theme 4: Patient emotions & intuition	
**Subthemes**	Age of diagnosis		First doctor approached				Prediagnosis testing				Sense of something not right	
	Symptoms prior to diagnosis		Specialists’ involvement prior to diagnosis				Screening				Emotional response	
	Early suspected diagnosis		Doctor who first identified WD				Diagnosis confirmation testing					
	Time frames from symptoms or clinical presentation to diagnosis		Explanation of diagnosis				Poking and prodding — excessive testing					
**Category 2: Multidisciplinary approach**												
	**Theme 1: Treatment management**					**Theme 2: Collaboration**				**Theme 3: Views on physician treatment**		
	Multiple specialists					Joint working				Positive		
	Time frames					Data sharing				Negative		
	Testing											
	Pregnancy											
	Lack of access											
**Category 3: Medication**												
**Theme 1: Types of medication**		**Theme 2: Side effects**			**Theme 3: Adherence**			**Theme 4: Cost of medication**		**Theme 5: Improvement**		**Theme 6: Ongoing symptoms and concerns**
**Category 4: The role of insurance**												
**Theme 1: Comprehensive insurance**							**Theme 2: Limited insurance**					
**Category 5: Education, awareness, and support**												
**Theme 1: Family support**				**Theme 2: Awareness in the medical community**					**Theme 3: Information sources**			

### Category 1: diagnosis journey

This category comprises 4 themes outlining the procedural aspects of the diagnosis experience as reported by both patients and physicians, in addition to the patient emotional experiences associated with them.

#### Theme 1: early presentation

The age at diagnosis varied widely (range 6–48 years); however, the patient’s age did not seem to influence their subsequent experience in terms of the speed of reaching diagnosis and the initiation of treatment. Patients presenting with predominantly psychiatric or neurological symptoms reported longer diagnosis journeys (range 1–16 years) than those presenting with hepatic symptoms or diagnosed through genetic screening (range 2 weeks-3 years). Presenting symptoms varied greatly (see Table 4). Some patients talked about early suspected diagnoses that included leukemia, thyroid issues, degenerative brain disease, and stroke.

**Table 4:** Codes in symptoms prior to diagnosis

Subtheme: Symptoms prior to diagnosis		
Physical/Biological	Neurological	Emotional/Psychological
• Liver issues • Fatigue or tiredness • Eye problems • Abnormal blood test results • Pain or bodily discomfort • Vomiting • Inconsistent menstrual cycle • Nosebleeds • Fever • Skin discoloration/jaundice • Eye problems • Swelling	• Tremors • Drooling • Dysphagia • Poor memory • Coordination or motor skills • Cognitive • Balance or gait • Speech problems • Dizziness • Weakness	• Affective disorders • Managing emotions • Other psychological issues

#### Theme 2: physician involvement and communication

The first doctor approached by patients for their presenting symptoms was generally a primary care physician; however, some patients reported presenting at the emergency room (ER) or via an allied healthcare professional (HCP) such as a speech therapist. Patients discussed seeing multiple specialists prior to receiving the diagnosis of WD (Table 5).

**Table 5:** Examples of physicians involved prior to diagnosis, as reported by patients

First doctor approached	Specialist involvement prior to diagnosis	Doctor who suspected WD
• Primary care doctor • ER doctor • Pediatrician • Speech therapist • First-year resident • Ophthalmologist • College health office • Cardiopulmonary specialist (family friend)	• Infectious disease doctor • Oncologist • Gastroenterologist • Nursing assistant • Ophthalmologist • Primary care doctor • Psychiatrist • Neurologist • Pediatric gastroenterologist • Intensive care staff	• Gastroenterologist • ER doctor • Primary care doctor • First-year resident • Pathologist • Ophthalmologist • Hepatologist • Cardiopulmonary specialist (family friend)

While some patients described receiving discouraging comments from their physician with regard to their prognosis and WD’s likely impact on their quality of life (e.g., ability to bear children), the physicians typically reported describing WD to their patients as something treatable but not something to be solved overnight.

*“I mean especially in children, I usually say, Well, we have a treatment, so we have a treatment that works pretty well, we can get you to take it and consistently take it and continue monitoring like you’re supposed to, show up for labs etc., then we can usually manage it.” Physician WD06, pediatric hepatologist*.

#### Theme 3: extensive testing

Patients described the burden of ongoing testing throughout their diagnosis journey and the constant “poking and prodding,” including investigatory blood tests, urine samples, eye tests, procedures (i.e., surgery or biopsy), and imaging (i.e., magnetic resonance imaging or computed tomography scans). Physicians were consistent in their report of the tests employed to confirm diagnosis, specifying ceruloplasmin tests, 24-hour urine copper, and an eye exam for Kayser-Fleischer rings. All physicians reported that patients undergo genetic testing as part of the diagnostic process.

*“The pediatric GI [gastroenterologist] person will send a bunch of additional labs and find out the ceruloplasmin’s low, then they’ll send them to us, we’ll do the biopsy….” Physician WD06, hepatologist*.

#### Theme 4: patient emotions and intuition

Those patients diagnosed as children described a parent who pushed for tests due to an intuition that there was a deeper problem not yet identified by medical professionals. When receiving the diagnosis, some patients reported feeling negative emotions, such as being afraid. In contrast, others reported feelings of relief to finally receive an explanation as to what was wrong, following a complex journey.

*“It was just a weight off my shoulders, finally knowing what was wrong, and there was an answer to what was happening. It was like when it’s all raining and all of a sudden, the sun opens up and the clouds clear and you can see the sky; that was basically what it felt like.” Patient WD12, man, 19*.

### Category 2: multidisciplinary approach

This category contains data on the ongoing management of WD, as experienced by patients with WD and physicians managing them.

#### Theme 1: treatment management

Physicians emphasized the importance of a multidisciplinary team including hepatology, neurology, and psychiatry and supported by social work, physiotherapy, and psychology to manage care after diagnosis. Patients similarly reported engagement with gastroenterologists or hepatologists alongside their primary care physician but did not typically receive support from psychiatry or wider allied HCPs such as physical therapists. Some patients described a lack of access to WD specialists due to geographical distance.

*“All of those providers would be optimal, even in terms of a one-time assessment to understand that they do or do not need additional assistance. So really a hepatologist, neurologist, psychiatrist, and…or physical therapist to have a more holistic evaluation of their needs would be optimal.” Physician WD05, neurologist*.

Regular testing was discussed by both patients and physicians as a large part of WD management. Physicians emphasized the importance of specific tests, such as liver function, ceruloplasmin, and 24-hour urine copper tests. One physician talked about the difficulties a patient had accessing these tests due to geographical distance.

*“I’ve had one that I’m really struggling with that 24-hour urine collection…the mom really pushed out to at least yearly. But I was doing, every six months, ideally, I would want a 24-hour urine collection, and his hepatologist was recommending every three months.” Physician WD07, neurologist*.

Two patients had 2 successful pregnancies each, despite 1 patient being told that it would not be possible. The patients outlined the changes in management during this time, such as increased monitoring and meeting frequency.

*“I knew that penicillamine could cause disruption in collagen formation, so I knew that if I had a C-section, it might not heal as well, that’s why they dropped down my dose, for that reason. I was able to breastfeed.” Patient WD11, woman, 52*.

Some of the physicians discussed the additional monitoring and professional involvement required, such as a perinatologist and a high-risk obstetric clinic, alongside promoting good adherence to the low-copper diet and having the patient take zinc and avoid chelators.

*“I think ideally we’d talk with our patients of childbearing potential about that and make some decisions about treatment based on that, and so zinc is probably a safer agent, and so getting someone on zinc and getting someone stable on that would be ideal before they get pregnant. I think most of our Wilson’s patients we suggest being cared for by a high-risk OB/GYN [obstetrician-gynecologist], and then again it just depends a little bit on distance.” Physician WD04, hepatologist*.

#### Theme 2: collaboration

Most physicians discussed the importance of co-managing patients with professionals from other disciplines. Some physicians described challenges with local HCPs. Patients had mixed experiences; while the majority were aware that their physicians were working together and communicating, some felt the different HCP involvement was too isolated. Physicians, in particular those from COEs, believed that shared electronic medical records among HCPs supported multidisciplinary collaboration.

*“So, one of the things is also to co-follow patients with the local gastroenterologist, so on one hand to empower them, but it becomes sometimes very, very tricky to do so.” Physician WD01, hepatologist*.

#### Theme 3: views on physician treatment

Patients expressed varying opinions on their physicians. Most of the patient respondents had positive views of their physicians, describing them as being supportive, providing good advice on adherence to diet and medication, and managing side effects.

*“He was a very good doctor, he explained everything. And then I remember leaving the room and I’m guessing they talked seriously with my parents about the impact that it would have on my life and on their life.” Patient WD05, woman, 20*.

Some patients described more negative experiences, such as impersonal interactions where they did not feel heard, in addition to receiving discouraging comments around the time of their diagnosis. This occurred both for those diagnosed decades ago and those diagnosed more recently.

*“Well, he said I might die — he said by my next birthday, I’d be dead in a year. He told me all his patients had died — so he told me by my next birthday, I’d be dead by 20 years old, but I sure fooled him.” Patient WD08, man, 73*.

### Category 3: experience with medication

Medication was discussed as a crucial part of management for WD; both patients and physicians described a range of experiences, outlined in the themes below.

#### Theme 1: types of medication

Zinc was widely discussed by both patients and physicians, with most of the patients solely using zinc, which required a regime that they described as “onerous.” Physicians recommended a combination of a chelator and zinc as the best practice, even for maintenance. Some patients and physicians also talked about their experience of experimental medication in clinical trials, antidepressants, and movement disorder medications.

*“The first medication I was on was tetrathiomolybdate… I was guinea pig Number 11.” Patient WD10, woman, 51*.

#### Theme 2: side effects

Half the patients recalled issues with side effects from medication. A commonly reported side effect was nausea and vomiting, relating to zinc. Side effects to chelators were reported, such as rashes, and 1 patient experienced a tightness in their throat. Similarly, physicians spoke about how zinc causes gastrointestinal upset. Physicians theorized this could be why some stable patients were using only a chelator because zinc was not well tolerated.

*“With taking a lot of zinc, you get a lot of stomach aches and once or twice a month you have serious gastro problems that include diarrhea. That is the most serious side effect of taking a lot of zinc.“ Patient WD04, man,19*.

#### Theme 3: adherence

Adherence to the medication and dietary regimen was described as challenging, by both patients and physicians, particularly in childhood. Some methods such as pill boxes, alarms, and printouts of high-copper foods were offered by physicians; however, some patients said they were not offered support for adherence challenges. Physicians also discussed using urine and blood tests to monitor adherence. Some physicians suggested that non-adherence to medication was due to their side effects, with 1 also highlighting cases whereby loss of insurance due to life events such as job loss also contributed.

*“…trying to get them to adhere to a low-copper diet and take their zinc every day or their trientine…there’s people that scrap this…It can be difficult, I’ve had… teenagers in college and their labs are completely out of whack, and they swear they’re taking their medication.” Physician WD06, pediatric hepatologist*.

#### Theme 4: cost of medication

Patients understood the cost of medication, particularly chelators, and discussed the barriers this presented. Generally, physicians viewed zinc as a cheap and accessible medication option for all patients. Physicians acknowledged that chelators, such as trientine, are not affordable to many patients, which perhaps explains why only half of the patients interviewed were using them. One patient expressed fear about the potential prohibitive costs of new treatments when they reach the market.

*“I am not taking penicillamine, I am not taking trientine. I guess I could get funding for that. If I don’t have funding for that, those medicines are real expensive. Basic things that are cheaper.” Patient WD03, woman, 53*.

#### Theme 5: improvement

Many patients experienced improvement in their symptoms due to the medication, including reduction in liver cirrhosis or tremors. The most reported improvement was the disappearance of Kayser-Fleisher rings in the eyes.

Patients also reflected on how the improvements made them feel, and how they were able to progress in their life despite having WD. For a few patients, they were also able to reduce their medication once symptoms had improved.

*“… in 14 years, my liver has not had any more cirrhosis because of my medicine.” Patient WD05, woman, 20*.

#### Theme 6: ongoing symptoms and concerns

Some patients reported a range of ongoing symptoms, some still linked to their initial presentation and others that had emerged since diagnosis. The most common ongoing symptoms were tremors, fatigue, and depression/low mood. However, the reason for these ongoing symptoms and their relationship to the patient’s medication regimen are unclear.

Neurologists spoke about the ongoing management of movement-related symptoms and stressed that patients can still experience some of these manifestations despite being on the optimal treatment for the movement disorder.

*“If people have more manifestations, kind of a combination of the things we talk about tremor or Parkinsonism, dystonia, those people have a tendency to get better if they’re on medicine, but they might also continue to have residual symptoms, even with the optimal treatment after time, and then people who are severely affected often remain severely affected, even with treatment.” Physician WD04, neurologist*.

### Category 4: influence of insurance

Participants described a range of insurance providers from private insurance through family and work, to federal health insurance programs for those with lower income or disability.

#### Theme 1: Comprehensive insurance

Some patients spoke about their insurance covering a large part of their tests and treatment for WD. Patients who reported having private or comprehensive insurance tended to be on chelators, whereas patients with less comprehensive insurance were on zinc.

*“It covers everything…They started me with penicillamine because it was a lot less money; the medicine I’m on now is $50,000 a month per my insurance, and so they didn’t want to put me on that immediately without trying penicillamine first.” Patient WD05, woman, 20*.

#### Theme 2: limited insurance

Patients reported not getting tests, treatments, or prescription zinc and chelators due to limited insurance. All physicians talked about experiencing insurance barriers, both for approving chelation therapy and prescription zinc.

*“It is good that I have insurance, but X is the worst insurance if you are American because it is for lower income. They are helping me with the basic things, but since that insurance isn’t very good, they are not helping me with more expensive [things]…” Patient WD03, woman, 53*.

### Category 5: education, awareness & support

This category comprised elements of support and knowledge that both patients and physicians believed contributed to the good clinical management of WD.

#### Theme 1: family support

Patients spoke about the various ways their family had supported them throughout their journey, from advocating with physicians to offering support with the medication regimen and dietary restrictions, particularly those who were diagnosed in childhood. Physicians also acknowledged that family support is as important as having good insurance. One physician stated that family support was particularly helpful in cases where there is non-adherence to medication and where more severe presentations involve neurological issues.

*“Well, we try, education is a big part of it, and getting the family, if there is family and friends to be part of the equation for trying to get someone compliant.” Physician WD04, neurologist*.

#### Theme 2: awareness in the medical community

Patients spoke about encountering HCPs at all stages of their journey who knew little to nothing about WD. The physicians interviewed were not surprised by this due to the rarity of the disease and most HCPs’ lack of exposure to it. They emphasized the importance of having a WD specialist involved in some way in the management of patients with WD.

*“…because it is a rare disease…it needs to be brought to attention that there are things out of the norm, and people have these situations. Doctors are busy; they can’t think of absolutely everything, but the awareness does kind of need to be there.” Patient WD08, woman, 40*.

#### Theme 3: information sources

Patients discussed drawing on a variety of sources for information and education. This included Facebook groups and rare disease websites and forums; however, 1 physician expressed some concerns over the reliability of these sources. Some patients reported their physician as the principal source of information. But physicians did feel there was a need for more information, with 1 stressing the need for patients to self-educate and self-advocate.

*“We certainly point people to the Wilson’s Disease Association for education and support, and it’s often a good way for them to continue to read about the illness and connect with other patients because it’s a rare condition and being part of that community can often be helpful in terms of understanding where they are and not being on their own as much.” Physician WD05, neurologist*.

## Discussion

There is a lack of qualitative patient-reported experience data for WD in the US. This current study is the first to explore and compare the patient and physician experiences of multidisciplinary management for WD using semi-structured qualitative interviews. The physician sample was recruited via COEs, so the physicians’ views and experiences reflect best practice in clinical care in the US. The patients interviewed were accessing a range of hospitals (COEs and local academic and community hospitals), with only an estimated 20% of the sample being seen in a COE.^1^ This difference should be considered when comparing patients’ views and experiences.

The patients reported that the time to reach a diagnosis from an initial presentation was considerably longer (up to 16 years) than the physicians’ optimal time frame (3 months). Other research suggests that diagnosis should be as soon as possible to reduce the risk of liver injury and neurological damage [14–16]. Delays in diagnosis are well documented in the literature [12, 13] and are often related to diagnostic errors. A recent survey of 151 Italian WD patients revealed 55% were misdiagnosed [17]. Misdiagnosis was similarly seen in our qualitative sample, with many of the patients experiencing a range of suspected diagnoses before they were diagnosed with WD. The psychiatric manifestations of WD are frequently misdiagnosed as primarily mental illness [18, 19], which was reflected in the current sample, where patients who described some psychiatric issues in their initial presentation tended to experience a longer time to diagnosis. It is thought the average time between the onset of psychiatric symptoms and diagnosis of WD is 2.4 years [20]. The new AASLD guidance now recommends that psychiatric evaluation is essential for any patient with WD presenting with psychiatric or neuropsychiatric features of WD, and consideration of counselling and/or psychotropic adjunct treatments [8]. Where neurological symptoms are present, the new guidance also recommends a multidisciplinary approach, involving speech therapy for dysarthria and dysphagia, as well as physical therapy and occupational therapy [8].

In the current study, some patients described the difficult medication regimen, especially for zinc. In a recent survey of 21 WD patients in the USA and Canada, one of the most burdensome aspects of the illness reported was the medication regimen [21], which is one of the main reasons for poor medication adherence, along with zinc’s side effects, usually nausea [17] and gastrointestinal upset, an issue also reported in this sample. Poor adherence is commonly highlighted in the WD literature [6, 22] and is seen in around 40% of patients [15]. Most patients in our study described being provided advice/tools by their physicians to support adherence to their medication and easing side effects. The methods described to promote medication adherence (pill boxes, alarms, taking medication with a cold cut of meat to offset side effects) could be beneficial to local specialists and primary physicians elsewhere. Some studies have shown that interventions, including close multidisciplinary follow-up, can also support medication adherence [23, 24]. Less frequently mentioned in the literature is the importance of adherence to the low-copper diet; patients in our sample also described this as challenging. Access to a nutritionist to support those who may struggle with dietary change is essential, and in most cases was offered to patients in this sample. Additionally, ensuring patients have clear information about the low-copper diet and are aware that it is not only an essential first step for managing WD but also a lifelong commitment for maintaining health when asymptomatic.

The use of a chelator in addition to zinc and the low-copper diet is the standard recommended treatment for WD, according to both this sample of physicians and EASL and AASLD guidelines [2, 8]. Those patients who were not on a chelator in our study commonly identified insurance coverage as the barrier. Patients are aware of how costly chelation therapy is, and although zinc therapy alone can be adequate for some patients, for others who require rapid copper reduction or have poor tolerance to zinc, this inability to access chelators, principally trientine, can be extremely detrimental [25].

One patient participant had lost access to their prescription zinc; whether there is a difference between prescription and over-the-counter zinc garnered a mixed opinion in the physician interviews. A comparison study of the effectiveness of prescription zinc acetate and alternative zinc preparations typically found over-the-counter zinc (zinc salts), implied that both were effective, as the absorption is good in many but not all patients [22]. Like our study, the North American survey previously mentioned also found that access to medication was one of the most common concerns for adult patients’ future well-being [21]. While there are patient assistance programs aimed at increasing access to medication, these are not available to everyone [25]. HCPs outside of COEs could consider implementing examples of best practice; for instance, the authors are aware of the Yale New Haven Health outpatient pharmacy working with insurance companies to enable better access to medication, particularly for those on disability or with issues gaining insurance authorization.

Whilst there is conflicting advice regarding WD management during pregnancy in the literature [9–11], physicians in our sample had a good grasp on how to manage the medication of these patients in accordance with guidelines. Further suggestions feature in the new AASLD guidelines, including the recommendation for pre-conception counseling, genetic testing, and discussions of medication safety [8].

The North American survey did not identify emotional burden as a severe issue; however, when this topic was mentioned, the most frequent impacts described were feeling overwhelmed, anxious, or worried and having concerns about the future [21]. Our study had similar findings, with the most commonly reported emotional impacts by patients being feelings of concern about the future, in addition to relief in relation to the diagnosis journey. A German retrospective cross-sectional study of 68 WD patients revealed this population is at risk for major depressive disorders [26]. Many of the patients interviewed in our study described mental health difficulties, both pre and post diagnosis. It appeared that recommendations that patients be screened, examined, and offered information about depression [26] are largely being implemented, according to our sample. However, the uptake for psychological support was low. A unique finding in our study was the perceived burden of multiple testing throughout the diagnosis journey, as identified by both patients and physicians, as well as challenges for patients and physicians trying to access tests locally.

A recent Italian qualitative survey study found only 19% of the respondents felt their primary care providers were able to grasp the disease [19], and this lack of confidence in local and primary HCPs was mirrored in our findings. Historically, WD patients have struggled to access adequate medical attention, due to a lack of clinician knowledge and the rarity of the condition, resulting in delayed diagnosis [13, 16]. The degree of awareness in the medical community can affect patient satisfaction with the patient-physician interaction, particularly in rare diseases, where patients often become an expert in their disease and seek information from sources other than their physician [27].

The current study highlights that access to adequate medical treatment is still an ongoing issue for this patient population and thus requires a call for action; there is a need for more specialists and more education and awareness outside of COEs. A focus on the wider medical community and for local physicians (particularly gastroenterologists) to understand the complexities of WD and the monitoring it requires would be beneficial. Physicians’ (including both specialists and general primary care) access to reliable, up-to-date information could result in better prognoses for patients, in addition to improved communication strategies and referral pathways into specialists. It is hoped the new AASLD guidance, signaling the need for a multidisciplinary treatment approach by the AASLD, will serve as a catalyst for this education. A few participants in this sample felt only their families cared about them, and the physicians emphasized the importance of the caregiver role. In particular, neurological presentations whereby patients can experience involuntary movements such as dysphagia, drooling, dysarthria, and gait and posture disturbances can create strain on caregivers [28]. In another advancement, the new AASLD guidance now suggests that patient counseling include assessment of the strain on parents and caregivers [8]. Both patients and physicians agreed that a diagnosis of WD can be an adjustment for the whole family. Physicians can also direct patients to up-to-date information and emotional support to empower them and their caregivers.

### Study limitations and future directions

A note of caution about the definition of COE, which relies exclusively on the assessment of a patient association. The patient sample was diverse in terms of age, gender, age of diagnosis, and insurance coverage, but was not heterogeneous in terms of race, with most of the sample being White (83%), which does not reflect the distribution of WD across ethnic groups [4, 21]. Therefore, our study cannot be generalized across all US communities, and further research is required to explore how the issues identified in our research impact on different races and ethnicities.

This study has generated questions that can be explored further in a quantitative study. Factors that potentially influence access to medication and WD specialists such as insurance coverage and geographical proximity were identified in our study, but to understand how they affect health outcomes would require a longer-term follow-up using a larger and more diverse sample.

## Conclusions

This qualitative study of the healthcare experience of patients with WD and physicians managing WD in the US has shown the importance that both patients and physicians place on the coordination of care among several specialties to manage this complex condition. Patients can face a long diagnosis journey, particularly when psychiatric symptoms are present, and steps can be taken to reduce challenges women face during pregnancy. Comprehensive care may be facilitated by easy access to reliable and up-to-date information for both physicians and patients across all geographic locations, and this could be led by COEs. Support for managing adherence to medication and side effects appears to be consistently required, as does family support for adolescent patients. Healthcare inequity prompts the need for more community outreach and education. This study highlights concepts for quantitative exploration in a larger and more diverse sample, namely the health outcomes for patients without access to WD specialists outside of COEs and for patients who have psychiatric symptoms, who have poor insurance coverage, and who represent diverse racial or ethnic backgrounds.

## Methods

A cross-sectional qualitative study design was used, in which primary data were collected from one round of 1:1 interviews with adults receiving care from a physician in a US hospital/clinic for the management of WD and with physicians working in a hospital/clinic managing patients with WD in the US. Ethical approval was received from the Salus Institutional Review Board (Ref no: ALEX2020).

Two of the authors (PH & UT) formed the Research Steering Group (RSG). The RSG provided input into the study design, informed the data analysis, and shared advice on medical terminology via 2 video-conference sessions, once after the codebook was developed from the patient interviews and again after it was updated following the physician interviews. The RSG also attended an initial theme review once all the data had been analyzed.

### Participants

Patient participants were eligible for interview if they were aged 18 years and older living in the US; had a clinical diagnosis of WD; had sufficient English to participate; were willing and able to participate in a 60-minute telephone interview; and were willing to provide oral or written informed consent.

Physicians were eligible for interview if they were board-certified neurologists, gastroenterologists, or hepatologists who have direct responsibility for the management of patients with WD, practice in a hospital/clinic in the US, and had managed patients with WD in the last year.

### Data collection

A target sample size of 20 (12 patients and 8 physicians) was proposed, considering the rarity of the disease, the specialists who manage it, and the geographical spread of the potential participants. Sample sizes in qualitative research are largely determined by the heterogeneity of the total study population, the ability to reach saturation (i.e., the point when no new analytical information arises anymore, and the study provides maximum information on the phenomenon), and pragmatic reasons such as rarity of the disease, geographical spread of source population and study timelines. Previous research has documented that 12 interviews are sufficient for reaching theme saturation within a homogenous sample [29].

Patients were recruited via a specialist recruitment agency, Global Perspectives, using a combination of internal databases and social media, between 3 February and 12 April 2021. A sampling matrix was provided to support the recruitment of a diverse sample according to patients’ time since diagnosis, ethnicity, gender, and insurance status. Physicians were identified via referrals from Alexion, Astra Zeneca Rare Disease; referrals from the physicians participating in the RSG; and a snowballing technique wherein recruited physicians were asked to recommend a colleague. Physicians were approached directly by the research team via email between 27 April and 21 July 2021 and provided with a study information sheet. Debarment checks were conducted prior to conducting the interview to comply with The Sunshine Act [30]. Following participants’ completion of written informed consent, telephone interviews (60 min) were conducted with 12 patients and 7 physicians by a trained qualitative interviewer from the research team (NS) using semi-structured discussion guides developed from a targeted literature review. The discussion guide covered patients’ experience of pre-diagnosis, diagnosis, initial treatment, and ongoing management and physicians’ reflection of patient opinions, symptoms, diagnosis, management within physician specialty, and multidisciplinary team management.

All interviews were audio-recorded and transcribed verbatim, with all identifying information removed from the transcripts prior to analysis. Half the patient interviews were completed before the physician interviews were commenced. This allowed the research team to summarize key topics from the patient interviews and incorporate them into the physician interviews. Honoraria payments of $125 for patients and $250 for physicians were offered for participants’ time.

### Analysis

Transcripts were uploaded to NVivo (v12) [31] for data management and thematic analysis [32]. Figure 1 outlines the analysis process. The following steps were taken:

**Figure:**
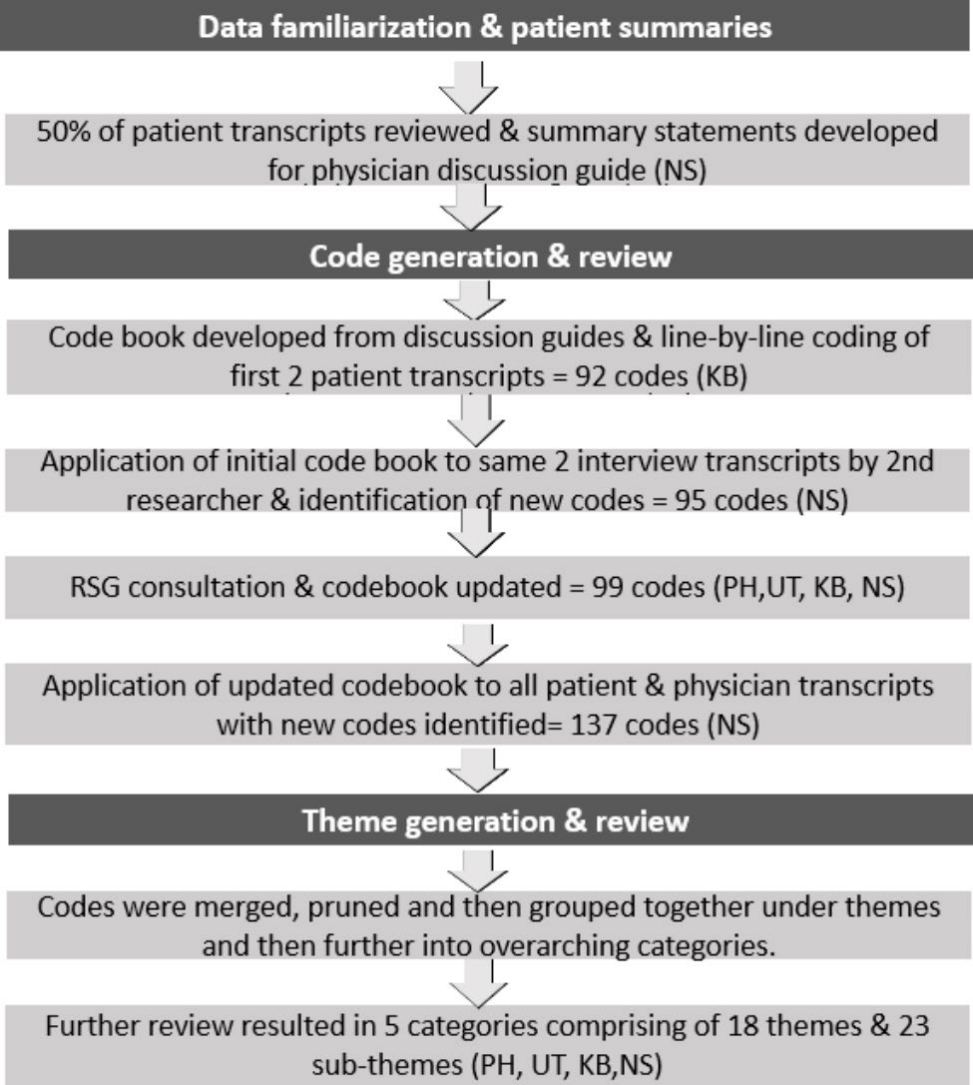

#### Data familiarization and patient summaries

Two researchers (NS and KB) read through the transcripts in chronological order to become familiar with the data, noting initial ideas for codes and reflections and thoughts to support the analysis.

NS developed patient summaries from the first 6 transcripts and a set of 6 statements for inclusion in the physician discussion guide.

#### Code generation and review

The codebook was derived by first drawing on the patient interviews and subsequently revised upon the introduction of the physician transcripts. One researcher (KB) developed the first draft of the codebook from a review of the discussion guides and line-by-line coding of the first 2 patient transcripts. A second researcher (NS) then applied this codebook to the same 2 patient transcripts, and a meeting was held to discuss any disagreements with codes or suggestions for new codes, followed by a discussion with the RSG. The codebook was then applied to the remaining patient transcripts by NS and then to the physician transcripts. The codes underwent further revisions following the coding of the physician transcripts, and then this revised codebook was further discussed with the RSG, resulting in a final codebook of 137 codes.

#### Theme generation and review

Theme identification was an iterative process starting from the generation of the early draft of the codebook. As codes were merged, pruned, and added, codes were then grouped together under themes and then further into overarching categories.

The emerging sets of themes and categories were developed by NS and KB jointly through a series of meetings. A meeting with the RSG largely confirmed the categories, but the RSG suggested some regrouping of themes and subthemes. The final analysis resulted in 5 overarching categories comprising 18 themes.

Interrater reliability was assessed twice using the function in NVivo and showed that on average consensus was reached for > 80% of codes.

A theme saturation exercise was completed following all analysis, by counting the occurrence of each theme per chronologically ordered interview. Saturation was reached by the fourth interview, with no new themes established beyond this point.

## Data Availability

Not applicable.

## Abbreviations

AASLD: American Association for the Study of Liver Diseases
COE: Center of Excellence
EASL: European Association for the Study of the Liver
ESPGHAN: European Society for Paediatric Gastroenterology Hepatology and Nutrition
GI: Gastrointestinal/Gastroenterologist
HCP: Healthcare professional/provider
RSG: Research Steering Group
US: United States (of America)
WD: Wilson disease

## Footnotes

1 COEs are defined and designated by the Wilson Disease Association. See https://wilsondisease.org/living-with-wilson-disease/centers-of-excellence/.
